# Supplementary material for: Analysis of Molecular Networks in the Cerebellum in Chronic Schizophrenia: Modulation by Early Postnatal Life Stressors in Murine Models
Source: Int J Mol Sci. 2021 Sep 17;22(18):10076. doi: 10.3390/ijms221810076 (PMC8469990; doi:10.3390/ijms221810076)
Supplement: Supplementary file 1 [file ijms-22-10076-s001.zip › Supplementary data/Figure S1.pdf]

Figure S1

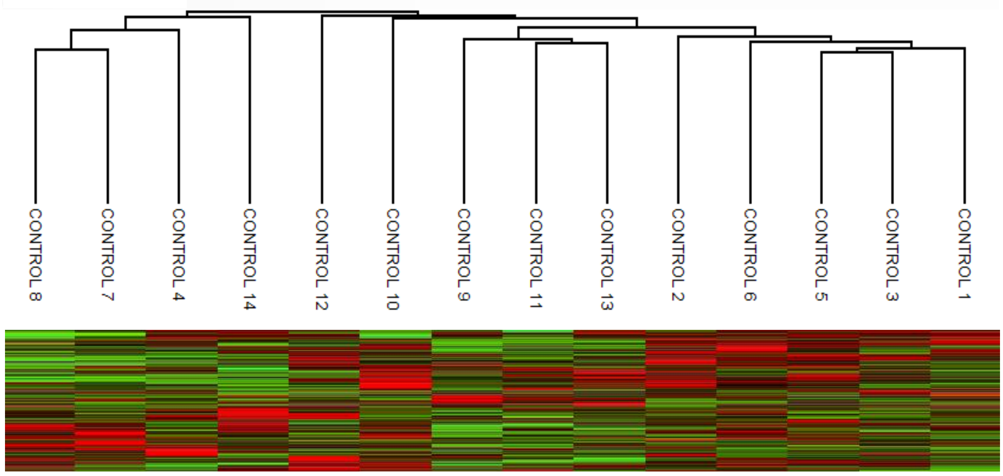

	C8	C7	C4	C14	C12	C10	C9	C11	C13	C2	C6	C5	C3	C1
Gender	M	M	M	M	M	M	M	M	M	M	M	M	M	M

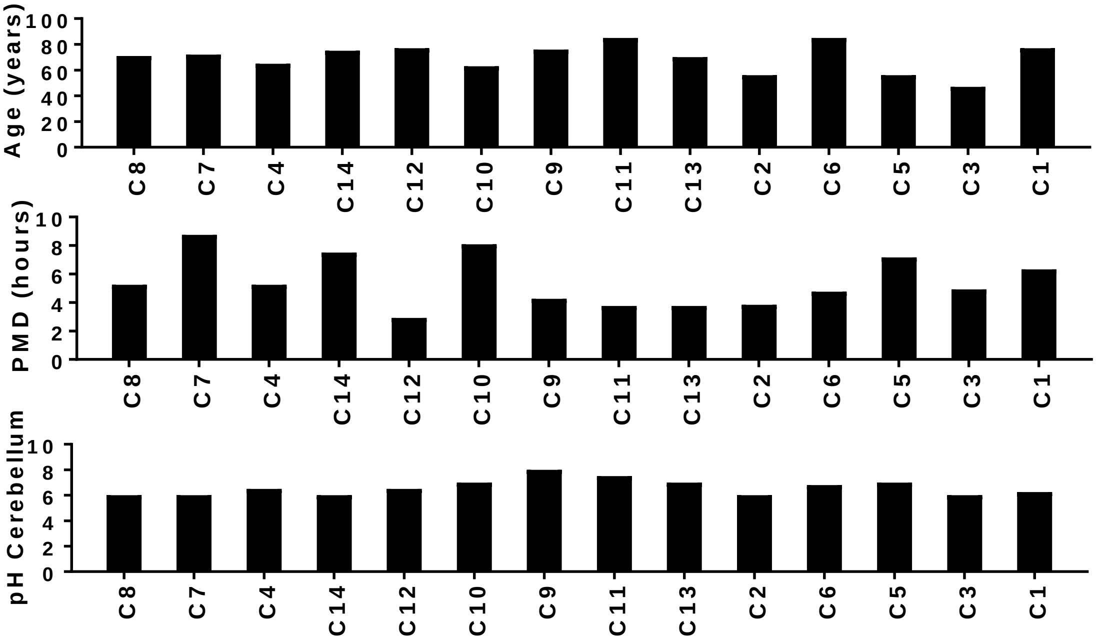

Figure S1: Unsupervised hierarchical clustering analysis for quantified proteins from *postmortem* cerebellum of 14 healthy control samples. The table below it shows the gender. The graphs show the tissue-related features of each healthy control sample. M: Male, PMD: *Postmortem* time delay.
