# Supplementary material for: Analysis of Molecular Networks in the Cerebellum in Chronic Schizophrenia: Modulation by Early Postnatal Life Stressors in Murine Models
Source: Int J Mol Sci. 2021 Sep 17;22(18):10076. doi: 10.3390/ijms221810076 (PMC8469990; doi:10.3390/ijms221810076)
Supplement: Supplementary file 1 [file ijms-22-10076-s001.zip › Supplementary data/Figure S2.pdf]

Figure S2

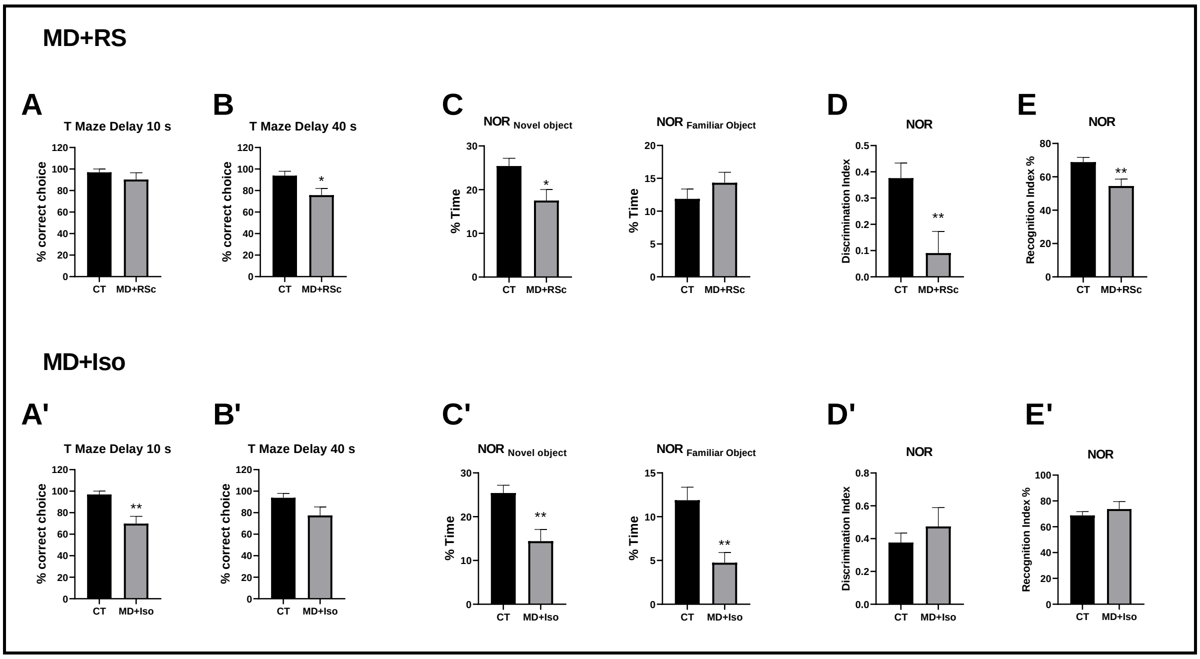

Figure S2. Behavioral analyses of double-hit murine models for schizophrenia. Percentage of correct choices in alternation task T-maze test in rat tested at 10 (Figure A,A') and 40 (Figure B,B') s delay intervals. Percentage of time (s) exploring novel or familiar object (Figure C,C'), discrimination (Figure D,D') and recognition (Figure E,E') index in NOR test. Bars represent means ± SEM. $*p < 0.05$, $**p < 0.001$ vs control (CT) group. Statistical analysis was performed using the Student's t-test for samples with normal distribution, and the Mann-Whitney U test was carried out for non-parametric distribution.
