# Supplementary material for: Analysis of Molecular Networks in the Cerebellum in Chronic Schizophrenia: Modulation by Early Postnatal Life Stressors in Murine Models
Source: Int J Mol Sci. 2021 Sep 17;22(18):10076. doi: 10.3390/ijms221810076 (PMC8469990; doi:10.3390/ijms221810076)
Supplement: Supplementary file 1 [file ijms-22-10076-s001.zip › Supplementary data/Figure S3.pdf]

Figure S3

A. Axonal guidance (CLASP1, MW: 160 kDa; MW: ß-actin 42 kDa)

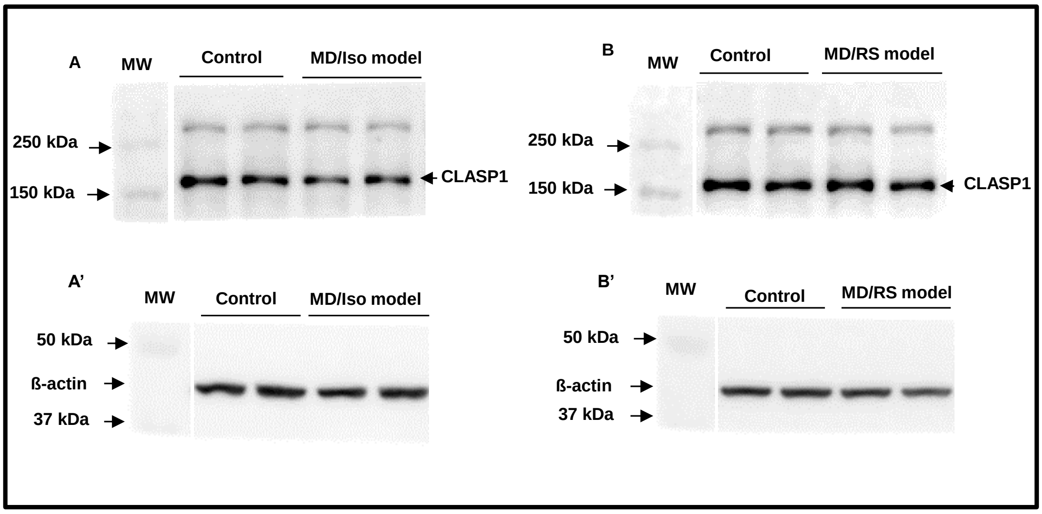

B. Vesicle-mediated transport (YWHAZ, MW: 28 kDa; MW: ß-actin 42 kDa)

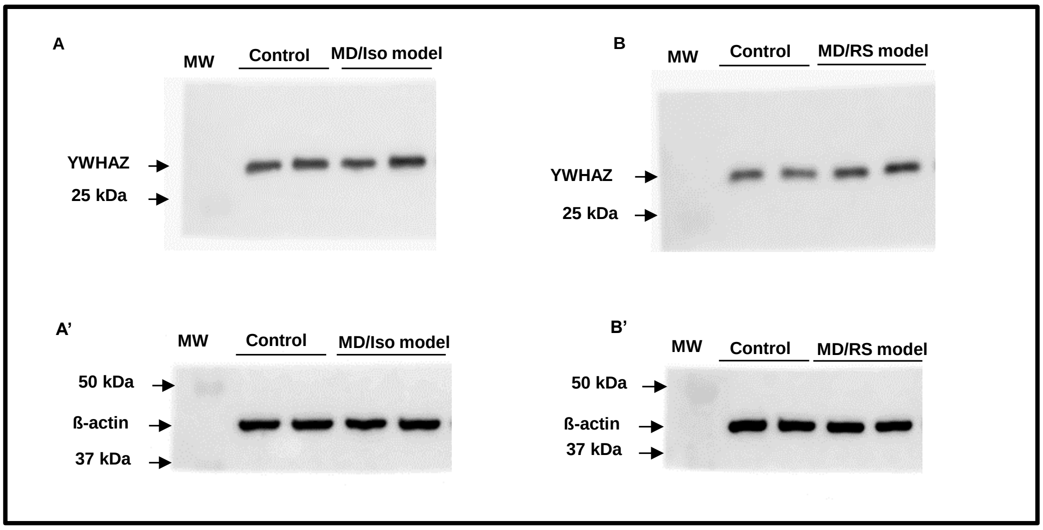

Continuation of Figure S3

C. TCA-Respiratory electron transport (NDUFB9, MW: 22 kDa; MW: ß-actin 42 kDa)

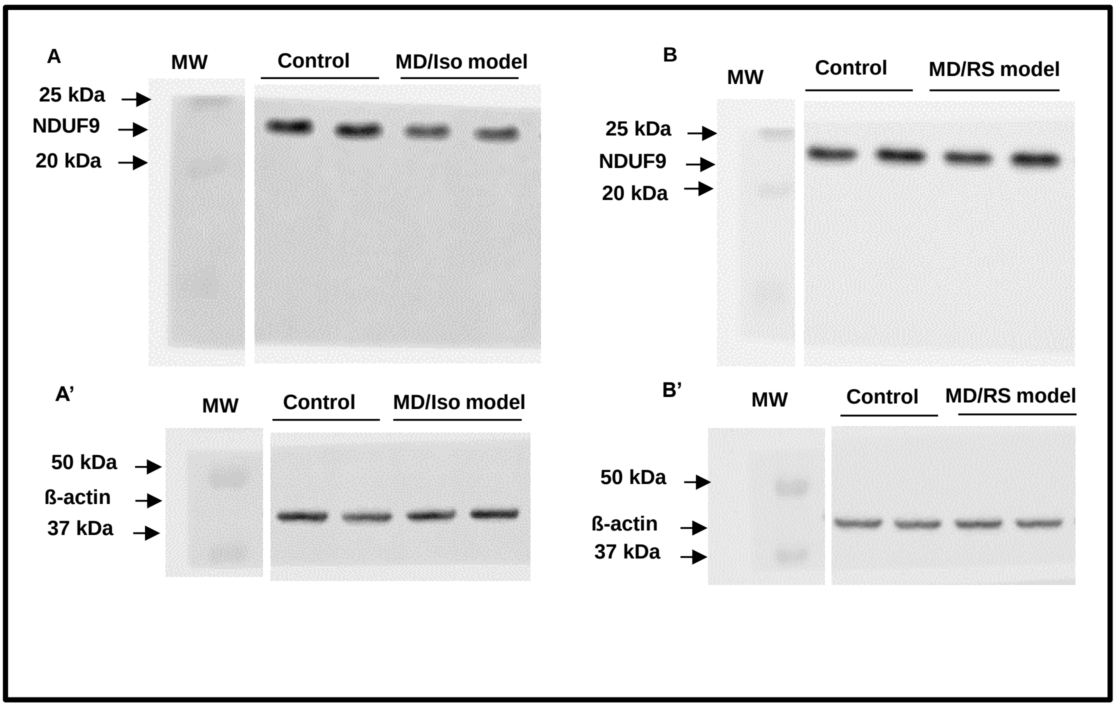

D. Neutrophil degranulation (METTL7A, MW: 28 kDa; MW: ß-actin 42 kDa)

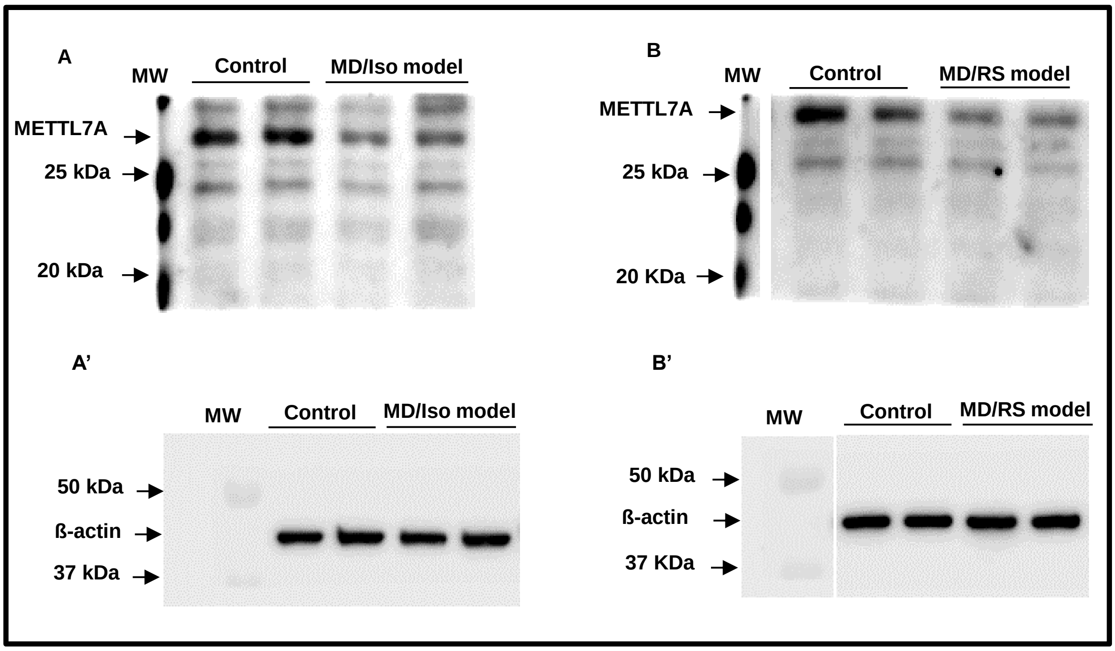

Figure S3. Validation of hit proteins in two double-hit murine models for schizophrenia. The hit proteins of the enriched pathways in our proteomic study were

analysed in two double-hit murine models for SZ**. Panel A**. Immunoblot of CLASP1, a hit protein from axon guidance. **A**. Expression of CLASP1 in the MD/Iso model in which it was significantly decreased in comparison with the control. **B**. Expression of CLASP1 in the MD/RS model; no significant changes were observed in this model. **A**' and **B'** show ß-actin expression used as a loading control. **Panel B**. Immunodetection of YWHAZ a hit protein from vesicle-mediated transport. In this analysis we did not observe any change in YWHAZ expression either in the MD/Iso or the MD/RS model in comparison with controls. **Panel C**. Immunoblot of NDUFB9, a hit protein from TCA/Respiratory electron transport. **A**. Reduced expression of NDUFB9 in the MD/Iso model in comparison with controls. **B**. Expression of NDUFB9 in the MD/RS model and controls. No significant changes were found in this model. **A**' and **B'** show ß-actin expression used as a loading control. **Panel D**. Immunoblot of METTL7A, a hit protein from neutrophil degranulation. **A**. Immunodetection of METTL7A in the MD/Iso model in which it was significantly downregulated in comparison with the control. **B**. Expression of METTL7A in the MD/RS model in which it was also significantly downregulated with respect to the control. **A**' and **B'** show ß-actin expression used as a loading control for METTL7A. **A**' and **B'** show ß-Actin expression used as a loading control. MD/Iso: maternal deprivation and isolation model. MD/RS: Maternal deprivation and restraint stress model. MW: Molecular weight. All protein levels were normalized to ß-actin values.
