# Supplementary material for: Analysis of Molecular Networks in the Cerebellum in Chronic Schizophrenia: Modulation by Early Postnatal Life Stressors in Murine Models
Source: Int J Mol Sci. 2021 Sep 17;22(18):10076. doi: 10.3390/ijms221810076 (PMC8469990; doi:10.3390/ijms221810076)
Supplement: Supplementary file 1 [file ijms-22-10076-s001.zip › Supplementary data/Supplementary Methods.docx]

**Supplementary methods**

***Postmortem* human brain tissue**

Samples from the cerebella of subjects with chronic schizophrenia (n=12) and healthy controls (n=14) were obtained from the neurologic tissue collection of the *Parc* *Sanitari* *Sant Joan de Déu* Brain Bank and the Institute of Neuropathology of the *Universitari de Bellvitge* Hospital respectively. All SZ subjects were institutionalized donors with long-term illness who had no history of neurological episodes. We matched SZ and control groups by gender (only male patients were included), age, *postmortem* delay (PMD) and pH (Table S1). Experienced clinical examiners interviewed each donor *antemortem* to confirm SZ diagnosis according to the Diagnostic and Statistical Manual of Mental Disorders (DSM-IV) and International Classification of Diseases 10. All deaths were due to natural causes. The study was approved by the Institutional Ethics Committee of Parc Sanitari Sant Joan de Déu. A written informed consent was obtained from each subject. The last daily chlorpromazine equivalent dose for the antipsychotic treatment of patients was calculated based on the electronic records of the last drug prescriptions administered up to death as described previously [1]. Human cerebellar lateral cortex was dissected from coronal slabs stored at - 80ºC, extending from the pial surface to white matter only including grey matter.

**Protein reduction, alkylation, LysC digestion, and desalting**

Protein extracts were prepared from tissue samples using NP40 lysis buffer as described previously [2]. Protein concentration was determined by Bradford assay (Bio-Rad, Hercules, CA, USA). 200 µg of total protein was lyophilized (Telstar, Lyoquest-55) per sample for mass spectrometry analysis. 200 µg of total protein extracts lyophilized from control and SZ lysates were resuspended in 100 µl 8M Urea, 50 mM Tris pH 8.2, 100 mM NaCl. The lysate was reduced with 3 mM dithiothreitol at 55 ºC for 30 min, alkylated with 20 mM iodoacetamide at room temperature for 30 min and quenched with additional 3 mM dithiothreitol for 30 min at room temperature. The extract was digested with the endoproteinase LysC at 10 ng/µl at 30ºC overnight. Peptides were desalting with OASIS MCX cartridge (Waters): cartridge was conditioned with Methanol, 5% ammonium hydroxide in Methanol, and 0.1% TFA; acidified peptides were loaded on the column and washed with TFA 0.1% and 0.1% Formic acid in Methanol, finally peptides were eluted using 5% ammonium hydroxide in Methanol. Eluted peptides were acidified with formic acid. and dried before re-suspending in MS Buffer composed of 3% formic acid, and 4 % acetonitrile in water.

**Liquid chromatography coupled to tandem mass spectrometry**

The LC/MS-MS analysis was performed in a Q-Exactive mass spectrometer (Thermofisher Scientific, CA, USA) coupled to an Easy nLC II liquid chromatography system. Peptides were loaded onto a 100 μm ID × 3 cm precolumn packed with Reprosil C18 3 μm beads (Dr. Maisch GmbH) and separated by reverse‐phase chromatography on a 100 μm ID × 30 cm analytical column packed with Reprosil C18 1.9 μm beads (Dr. Maisch GmbH). A gradient of 3% to 30% acetonitrile in 0.125% formic acid was used delivered at 225 nl/min over 130 minutes, with a total 180-minute acquisition time. Peptides were analyzed online on the orbitrap mass analyzer using a top 20 data-dependent acquisition with all MS spectra being acquired and stored in centroid mode. Full MS scans were acquired from 300 to 1500 m/z at 70,000 FWHM resolution with a fill target of 3E6 ions and maximum injection time of 100 ms. The 20 most abundant ions on the full MS scan were selected for fragmentation using 2 m/z precursor isolation window and beam-type collisional-activation dissociation (HCD) with 26% normalized collision energy. MS/MS spectra were collected at 17,500 FWHM resolution with a fill target of 5E4 ions and maximum injection time of 50 ms. Fragmented precursors were dynamically excluded from selection for 35 s.

**Data analysis**

Raw files were processed and analyzed using MaxQuant (version 1.5.3.8). MS/MS spectra were searched with Andromeda against UniProt fasta UP000005640 (Downloaded: 2015-06-30) with common contaminants added. The precursor mass tolerance was set to 7 ppm, and the fragment ion tolerance was set to 20 ppm. Search parameters included full LysC enzyme specificity with up to three missed cleavages permitted. The target-decoy database search strategy was used to guide filtering and estimate false discovery rates (FDR). Peptides matches were filtered to an FDR of ≤0.01. Proteins with at least one peptide were considered identified. Label-free quantification (LFQ) was selected for individual protein comparisons between control and schizophrenia groups. A quality cut-off for protein determination was the presence of the protein in at least 7 samples per group. The normalized LFQ intensity was referred to media of the controls. A significance value for each quantified protein was calculated using Student’s t-test and the correction of significance values of the quantified protein data set was performed following the Benjamini and Hochberg methods [3]. An FDR was computed for all significant values and the FDR threshold was set to 0.1.

The quantified proteins were imported into Perseus software platform (version 1.6.1.3) to check data quality obtained by LC/MS/MS and to visualize the data distribution[4]. We performed the following analyses: 1. Correlation matrix: the normalized LFQ intensity data were used to estimate a correlation coefficient matrix between controls and schizophrenia patients. For easy visualization, the correlation coefficient data were plotted as a heat map. 2. Hierarchical Clustering: This was carried out on Z-score transformed normalized LFQ intensity data for each protein using Euclidean distance.

**Murine models**

Three pregnant Wistar rats (Harlan Ibérica, Spain) at gestation days 14-16 were individually housed in a temperature/humidity-controlled environment in a 12h light/dark cycle with free access to food and water. The litter sizes varied between 7 and 11 animals. One was used as a control group and the others as part of a double-hit model. After birth, at postnatal day 9 (PD9), both double-hit litters were exposed to maternal deprivation for 24hrs as a first hit; this early stressful life event has an impact on prepulse inhibition in rats, similar to the alterations seen in schizophrenic patients. On PD21, the pups were weaned and one of the litters was exposed to isolation for 5 weeks as a second hit (PD21-56); although the pups were housed separately (1/cage), they could smell, hear, and see their siblings, but they could not come into physical contact with them. Following isolation, the animals were regrouped (DM/Iso). Meanwhile, the second hit for the other litter exposed to maternal deprivation involved restraint stress between PD 72 to 78 for 6 hours every day (DM/RS). These conditions represent suitable rodent models for the study of neuropsychiatric dysfunctions[5,6]. Group sample sizes were CT, n=11; DM/Iso, n=9; DM/RS, n=7. All experimental protocols adhered to the guidelines of the Animal Welfare Committee of the Complutense University in accordance with European legislation (D2010/63/UE). All efforts were made to reduce the number of animals used and minimize animal suffering in the experiments.

**Behavioral test**

**T-Maze:** Animals were exposed to an alternation task T-maze test [7]. This test is used to study how rodents operate with memory and spatial learning. The whole experiment was performed on a 140x80x40cm T-maze and consisted of three parts: habituation, training, and testing. For habituation, animals were placed on the T-maze with food spread in three five-minutes-rounds every day for two days. During the four days of training, animals received six trials a day. Each training trial consisted of two runs, a forced and a free run. In the forced run, rats were coerced to obtain a piece of food from the goal alley of the T-maze, with the other alley blocked by its door. Then, animals were placed back into the start arm for 10 and 40 s delay periods (three trials for each delay period). At the free run, animals were allowed to choose either goal alley. If the rats chose the same arm into which they had been forced, they did not receive a food reward, whereas if they chose the opposite arm, they received the compensation, and the choice was considered correct. The sequence of delays and forced-run food locations were randomized each day. It was considered that animals were trained when controls made more than 70% correct choices on two consecutive days. The testing phase was performed on the seventh day of the test. The pattern was similar to the one displayed in the training phase, with a 5-minute maximum intertrial interval. Rats were tested for their performance in the T-maze, recording the number of correct choices.

**Novel Object Recognition (NOR):** NOR detects cognitive and memory deficits. In this test, rodents can recognize and explore familiar and novel objects depending on the trial. It is performed in a 90x90x40cm stained box. The test consists of two different sessions: two days of preconditioning training followed by the day of the test. Rats are allowed to explore and recognize two similar objects during the preconditioning training sessions and gather information about these objects. In the test session, one of the familiar objects is replaced by a different novel object and rats are allowed to explore for five minutes. This session should show differences between the recognition of familiar and novel objects. For analyses, we determine the time in the familiar object in seconds (s), time in the novel object (s), and exploration time (s) using the ANY-MAZE Software. Moreover, we also calculate the percentage of time spent on familiar and novel objects during exploration, as well as discrimination index ((Time in novel object – Time in familiar object)/Exploration time) and percentage of recognition index ((Time in novel object/Time of Exploration)x100).

**Western Blot analysis**

Brain cerebellum samples were homogenized by sonication in PBS (pH=7) mixed with a protease inhibitor cocktail (Complete^®^, Roche, Spain). After determining and adjusting protein levels, homogenates of cerebellum tissue were mixed with Laemmli sample buffer (Bio-Rad, USA) and β-mercaptoethanol (50 µl/ml Laemmli), 15µg were loaded into an electrophoresis gel. Once separated on the basis of molecular weight, proteins from the gels were blotted onto a nitrocellulose membrane with a semi-dry transfer system (Bio-Rad) and were incubated with specific antibodies against: (1) Methyltransferase-like protein 7A (METTL7A, 1:750 in BSA 1%; ABclonal A8201); (2) NADH dehydrogenase (ubiquinone) 1 beta subcomplex, 9 (NDUFB9, 1:1000 in BSA 0,5%; sc398869, SCT); (3) cytoplasmic linker associated protein 1 (CLASP1, 1:1000 in BSA 0,5%; sc390159, SCT); (4) tyrosine 3-monooxygenase/tryptophan 5-monooxygenase activation protein zeta (YWHAZ, 1:1000 in BSA 0,5%; sc293415, SCT); (5) β-actin (1:10000; A5441, Sigma). Primary antibodies were recognized by the respective horseradish peroxidase-linked secondary antibodies. Blots were imaged using an Odyssey Fc System (Li-COR, Biosciences, Germany) and were quantified by densitometry (NIH ImageJ software). In all the WB analyses, the housekeeping protein β actin was used as a loading control, and each western blot was performed at least three times in separate assays. The data were presented as fold change from the control group.

**References**

1. Gardner DM, Murphy AL, O’Donnell H, Centorrino F, Baldessarini RJ. International consensus study of antipsychotic dosing. Am J Psychiatry. 2010;167(6):686–93.

2. Pinacho R, Villalmanzo N, Lalonde J, Haro JM, Meana JJ, Gill G, et al. The transcription factor SP4 is reduced in postmortem cerebellum of bipolar disorder subjects: Control by depolarization and lithium. Bipolar Disord. 2011;13(5–6):474–85.

3. Yosef BY and H. Controlling the False Discovery Rate : a Practical and Powerful Approach to Multiple Testing. J R stadtstical Soc. 1995;57;1:289–300.

4. Tyanova S, Temu T, Sinitcyn P, Carlson A, Hein MY, Geiger T, et al. The Perseus computational platform for comprehensive analysis of (prote)omics data. Nat Methods. 2016;13(9):731–40.

5. Bailoo JD, Varholick JA, Garza XJ, Jordan RL, Hintze S. Maternal separation followed by isolation-housing differentially affects prepulse inhibition of the acoustic startle response in C57BL/6 mice. Dev Psychobiol. 2016;58(8):937–44.

6. van Zyl PJ, Dimatelis JJ, Russell VA. Behavioural and biochemical changes in maternally separated Sprague–Dawley rats exposed to restraint stress. Metab Brain Dis. 2016;31(1):121–33.

7. Holloway T, Moreno JL, Umali A, Rayannavar V, Hodes GE, Russo SJ, et al. Prenatal stress induces schizophrenia-like alterations of serotonin 2A and metabotropic glutamate 2 receptors in the adult offspring: Role of maternal immune system. J Neurosci. 2013;33(3):1088–98.
