# Supplementary material for: Analysis of Molecular Networks in the Cerebellum in Chronic Schizophrenia: Modulation by Early Postnatal Life Stressors in Murine Models
Source: Int J Mol Sci. 2021 Sep 17;22(18):10076. doi: 10.3390/ijms221810076 (PMC8469990; doi:10.3390/ijms221810076)
Supplement: Supplementary file 1 [file ijms-22-10076-s001.zip › Supplementary data/Tabla S1.pdf]

Table S1: Demographic, clinical and tissue-related features of cases.

	Schizophrenia (n=12)	Control (n=14)	Statistic	*p*- value
**Gender**				
Male	100% (*n* =12)	100% (*n* =14)	N/A	N/A
Age (years)	72 ± 9	69 ± 11	78.5[a]	0.56
PMD (hours)	5.48 ± 2.29	5.46 ± 1.81	0.02; 25 [b]	0.98
pH Cerebellum	6.88 ± 0.49	6.61 ± 0.63	1.25; 25 [b]	0.22
**SZ diagnosis**				
Chronic residual	66.67% (n= 8)			
chronic paranoid	16.67% (n=2)			
chronic disorganized	8.33% (n=1)			
chronic catatonic	8.33% (n= 1)			
Age of onset of SZ (years)	22 ± 8	N/A	N/A	N/A
Duration of illness	50 ± 9	N/A	N/A	N/A
**Toxicology**				
Daily AP dose (mg/day) [c]	609 ± 507.10	N/A	N/A	N/A
First generation AP	16.67% (n=2)			
Second generation AP	58.33% (n=7)			
First and Second generation AP	8.88% (n=1)			
AP free	16.67% (n=2)			

Mean ± standard deviation; PMD, postmortem delay; SZ, schizophrenia; AP, antipsychotics; N/A, not applicable.

a Mann-Whitney U for non-parametric variables.

b T-statistic and degree of freedom for parametric variables.

c Last daily chlorpromazine equivalent dose was calculated based on the electronic records of drugs prescriptions of the patients as described (Gardner et al., 2010).
