# Supplementary material for: Analysis of Molecular Networks in the Cerebellum in Chronic Schizophrenia: Modulation by Early Postnatal Life Stressors in Murine Models
Source: Int J Mol Sci. 2021 Sep 17;22(18):10076. doi: 10.3390/ijms221810076 (PMC8469990; doi:10.3390/ijms221810076)
Supplement: Supplementary file 1 [file ijms-22-10076-s001.zip › Supplementary data/Table S2.pdf]

Table S2: Proteins previously reported in proteomic studies in schizophrenia

Cerebral area	Proteins previously reported in schizophrenia	References
**Cerebellum**	CAMK4; C11orf58; NDUFA12; AHCY; ERP44; PAFAH1B1; PRDX6; NCKAP1; LCP1; MYH9; NAXE; ACLY; CYB5R1; NAPA; CCT7; HSD17B4; BCL2L13; RAB21; RIMS1; UBE2I	Vidal-Domènech et al., 2020; Reis-de-Oliveira et al., 2020
**Anterior cingulade**	CS; GPD1; AHCY; CBR1; UCHL1; PGAM1	Clark et al., 2007
**Anterior cingulade cortex**	CS; ACAT2; SOD1;DDAH1;DCTN2; IDH2; HNRNPK; IARS; METTL7A; ACTR2; PPIA; LIN7A; PIP4K2A; NAPA; SPTAN1; SCRN1; TPM1; ARHGDIA; YWHAZ; YWHAB; COMT; HSD17B4; AP2M1; UCHL1	Clark et al., 2007; ClarK et al., 2006; Föcking et al., 2015; Martins de Souza et al., 2010; Smalla et al., 2008; English et al., 2011
**Anterior hippocampus**	UCHL1 ; DDAH1 ; SOD1 ; CAPZA2 ; YWHAE; YWHAZ ;PRDX6	Nesvaderani et al., 2009
**Posterior hippocampus**	PGAM1	Nesvaderani et al., 2009
**Anterior temporal lobe**	YWHAQ; YWHAZ; HSPD1; NDUFB5; SPTAN1; YWHAH; YWHAE; MYH9; PURA; HNRNPK; MOG	Saia-Cereida et al., 2017; Martins de Souza et al., 2009[b]
**Auditory cortex**	GNAQ; ATP1A3; UCHL1	Mc Donald et al., 2015
**Corpus callosum**	YWHAZ; UCHL1; MOG; NPM1; DDAH1; YWHAE; YWHAB; ANK3; SOD1; DDT; HP1BP3; HNRNPR; YWHAH	English et al., 2011; Saia-Cereida et al., 2015; Sivagnanasundaram et al., 2007; Saia-Cereida et al., 2016
**Dorsolateral prefrontal cortex**	DPYSL5; NDUFS1; HSPD1; SPTAN1; YWHAE; PGAM2; MOG; RAP2A; NDUFA12; GDAP1; NDUFB10; SIRPA; HP1BP3; DPYSL4; ADH5; GSTM3; IGSF8; CLSTN1; MAP6; PRDX6; TKT; ERP29; PGAM1; PURA; YWHAZ; CBR1; TCP1; HSPA9; BCL2L13; UCHL1; ARPC1A; ITGAV; SCRN1; GPM6A; MARCKS; TXN2; TPT1; CYB5A; RTN1; GNAQ; ATP5PD; PPA2	Saia-Cereida et al., 2015; Martins de Souza et al., 2009[a]; English et al., 2009; Martins de Souza et al., 2009[c]; Chan et al., 2011; Behan et al., 2009; Pinacho et al., 2016; Pennington et al., 2008; English et al., 2011; Wesseling et al., 2013; Novikova et al., 2006; Smalla et al., 2008; Prabakaran et al., 2004
**Hippocampus**	TPPP; UBE2M; YWHAE;SOD1; PRDX6; TCP1; UCHL1	Schubert et al., 2015; Föcking et al., 2011
**Mediodorsal thalamus**	YWHAZ; CBR1; SYN3; PGAM1; NDUFB9; TKT; MOG; HSPD1; PPIA; PITPNA	Martins de Souza et al., 2010
**Orbitofrontal prefrontal cortex**	RAB35; ATP2B3; PGAM1; RTN1; DDAH1; ATP1A1; MARCKS; MOG; YWHAH; SYN3; GPM6A; ATP1A3	Velasquez et al., 2017
**Wernicke's area**	DLD; PRDX6; PGAM1; NDUFS1	Martins de Souza et al., 2009[d]
**Insular cortex**	SNCG; ERLIN2; NPM1; COPS4	Pennington et al., 2008
